# Supplementary material for: Moonlight Is Perceived as a Signal Promoting Genome Reorganization, Changes in Protein and Metabolite Profiles and Plant Growth
Source: Plants (Basel). 2023 Mar 2;12(5):1121. doi: 10.3390/plants12051121 (PMC10004791; doi:10.3390/plants12051121)
Supplement: Supplementary file 1 [file plants-12-01121-s001.zip › Supplementary Figures.pdf]

The moonlight is perceived as a signal promoting genome reorganization, changes in protein and metabolite profiles and plant growth

Jeevan R. Singiri, Govindegowda Priyanka, Vikas S. Trishla, Zachor Agmon-Adler and Gideon Grafi*

French Associates Institute for Agriculture and Biotechnology of Drylands, Jacob Blaustein Institutes for Desert Research, Ben-Gurion University of the Negev, Midreshet Ben Gurion 8499000, Israel

Supporting information

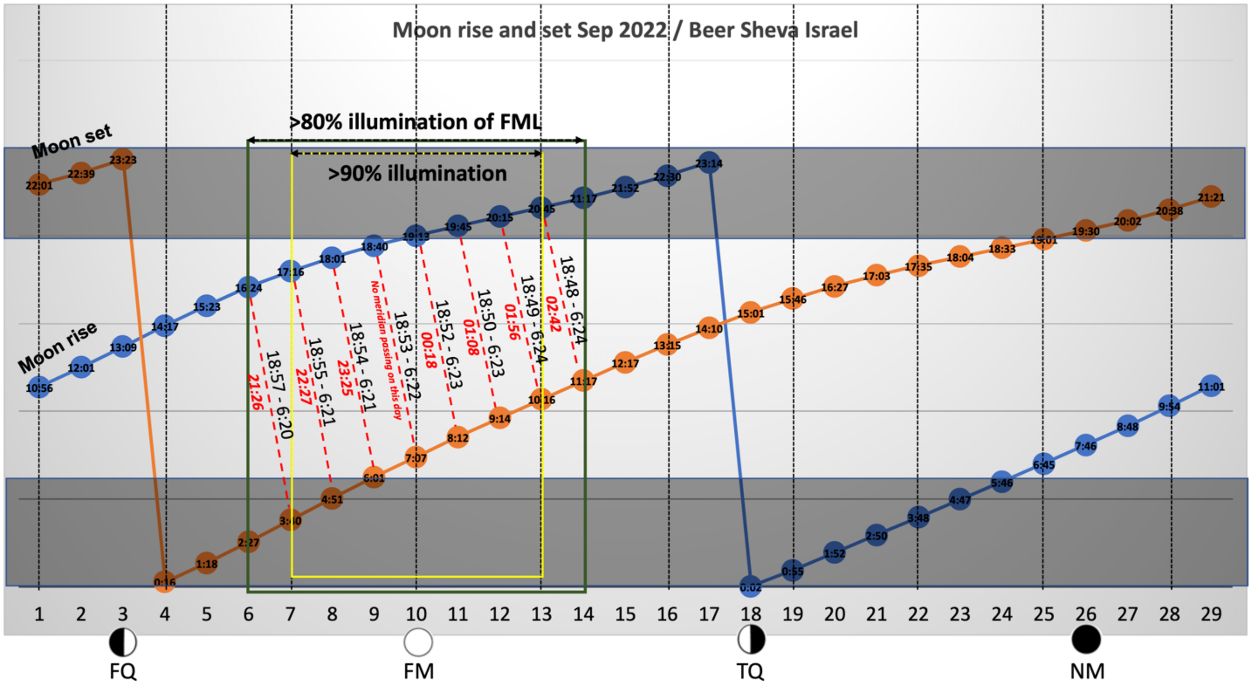

Fig. S1. The Moon rise and set during the month of September 2022 in Beer Sheva, Israel (data obtained from https://www.timeanddate.com/sun/israel/beersheba?month=9&year=2022). Green and yellow boxes represent the dates corresponding to moonlight illumination >80% and >90% of the full moon, respectively. Broken red lines indicate the moon rise (blue) and set (orange), the black vertical numbers refer to sun set and rise and red numbers to the time of meridian passing. FQ, first quarter; FM, full moon; TQ, third quarter; NM, new moon.

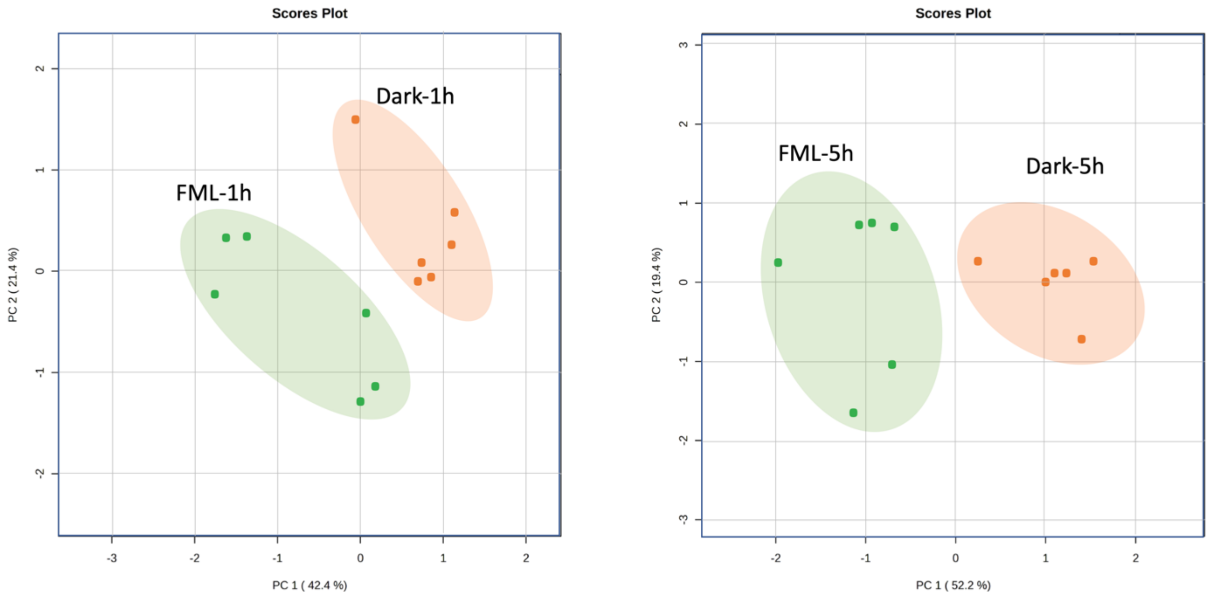

Fig. S2. A PCA score plot comparing all annotated metabolites (98 metabolites) extracted from of tobacco plant exposed for 1h (left panel) and 5h (right panel) to dark and FML.

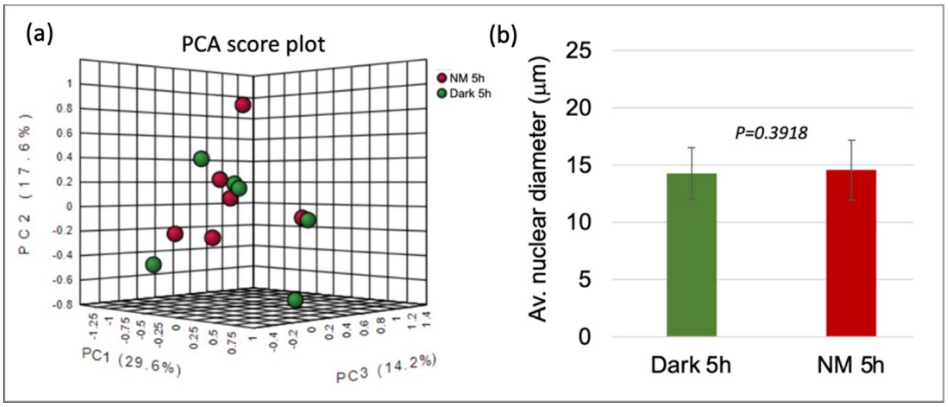

Fig. S3. Examination of light pollution at the experimental location. The experiment was run at the new moon (NM) stage with tobacco seedlings. (a) A PCA score plot comparing metabolite profiles after exposure for 5h to dark (Dark 5h) or to NM (NM 5h). (b) Average diameter of nuclei prepared from dark and NM-treated plants (n=100). Vertical bars represent the standard deviation. Statistical significance was performed by unpaired t test (GraphPad software).

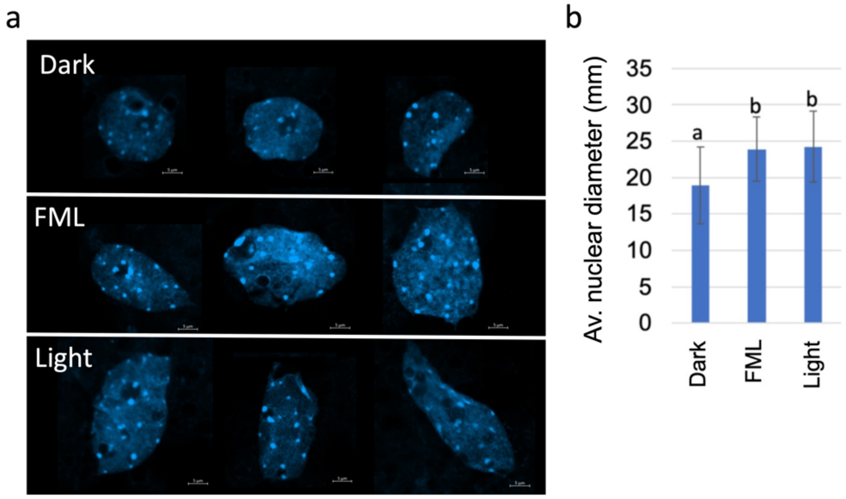

Fig. S4. FML and light induce increase in nuclear size/volume in *B. juncea* (Indian mustard) seedlings. (a) Leaves and cotyledons of mustard plants exposed to dark, FML or to light for 5h were fixed in acetic acid:ethanol (1:3), nuclei were prepared, stained with DAPI and visualized under a confocal microscope. (b) Average diameter of nuclei prepared from dark, FML, and light-treated plants (n=100). Vertical bars represent the standard deviation. Statistical significance was performed by a One-Way ANOVA Calculator, Including Tukey HSD (Social Science Statistics). Different letters indicate statistically significant differences between treatments ($p < 0.01$).

Nicotiana tabacum H3.3 accession # XP_016473270

MARTKQTARKSTGGKAPRKQLATKAARKSAPTTGGVKKPHRYRPGTVALREIRKYQKSTELLIRKLPFQR
LVREIAQDFKTDLRFQSHAVLALQEAAEAYLVGLFEDTNLCAIHAKRVTIMPKDIQLARRIRGERA-136

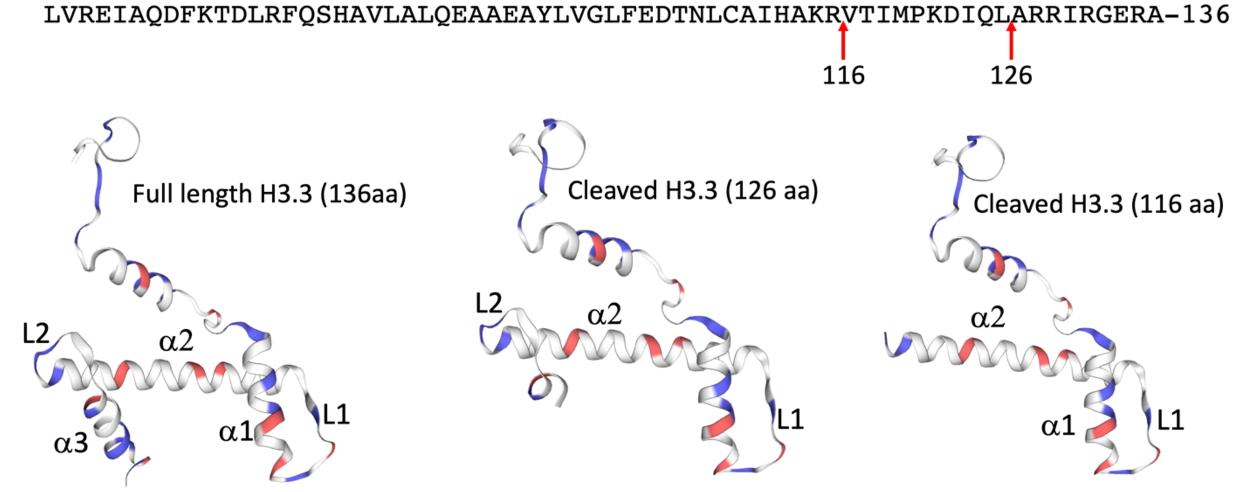

Fig. S5. Removal of 10 or 20 amino acids of H3.3 C-terminus results in loss of α3 helix. Protein modeling of tobacco histone H3.3 full length and C-terminal cleavage of 20 (red arrow 116) and 10 (red arrow 126) amino acids. Modeling was performed by the SWISS MODEL platform [50].
